# The role of ATP6V0D2 in breast cancer: associations with prognosis, immune characteristics, and TNBC progression

**DOI:** 10.3389/fonc.2024.1511810

**Published:** 2024-11-29

**Authors:** Jingyu Zhang, Lixian Yang, Bin Xu, Haibo Ji, Shuo Liu, Xiaohan Wang, Xiaolong Li, Quanle Wang, Zhenchuan Song

**Affiliations:** ^1^ Department of Breast Center, Fourth Hospital of Hebei Medical University, Shijiazhuang, Hebei, China; ^2^ Department of Breast Surgery, Xingtai People’s Hospital, Xingtai, Hebei, China; ^3^ Department of Breast Surgery, Fourth Hospital of Shijiazhuang, Shijiazhuang, Hebei, China; ^4^ Key Laboratory for Breast Cancer Molecular Medicine of Hebei Province, Shijiazhuang, Hebei, China

**Keywords:** ATP6V0D2, immune infiltration, breast cancer, prognosis, diagnosis

## Abstract

**Objective:**

Researches have identified ATPase H+ transporting V0 subunit d2 (ATP6V0D2) as a significant factor in various cancers. However, its prognostic value in breast cancer (BRCA) and its biological role in BRCA cells remain unclear.

**Methods:**

In this research, we examined the varying expression levels of ATP6V0D2 in both BRCA and normal breast tissue by utilizing information derived from databases including the Cancer Genome Atlas (TCGA) and the Gene Expression Omnibus (GEO), along with clinical samples. Survival studies were carried out to investigate the link between ATP6V0D2 levels and prognosis in BRCA patients. A series of enrichment analyses identified possible pathways associated with the differentially expressed genes in BRCA. The relationships among ATP6V0D2 expression, immune characteristics, and gene mutation were evaluated using Spearman’s test. Finally, the expression of ATP6V0D2 was identified by quantitative real-time polymerase chain reaction (RT-qPCR) alongside western blot analysis. Additionally, Cell Counting kit-8 (CCK-8), Colony formation, Transwell, Scratch healing, and Mouse xenograft tumor assays were conducted to assessed the impact of ATP6V0D2 knockdown on the biological functions in TNBC.

**Results:**

ATP6V0D2 exhibited high expression in a range of cancers and correlated with unfavorable prognosis in BRCA. Functional enrichment analyses revealed enrichment of extracellular matrix-receptor interaction, focal adhesion, and the signaling pathway of tumor growth factor-beta in the high ATP6V0D2 expression group. Additionally, ATP6V0D2 was closely associated with immune checkpoints. Its expression positively associated with the infiltration levels of macrophage and neutrophil, but inversely with CD8^+^ T and plasmacytoid dendritic cells. Mutation analysis revealed that PIK3CA, linked to decreased OS, exhibited a higher mutation rate in the ATP6V0D2 high expression group. Furthermore, ATP6V0D2 knockdown inhibited TNBC cells invasion, migration, and proliferation abilities.

**Conclusion:**

ATP6V0D2 acts as a promising indicator for both diagnosis and prediction of outcomes in breast cancer and could potentially be a novel therapeutic target for BRCA.

## 1 Introduction

Breast cancer (BRCA) stands as the most prevalent malignancy globally and the primary cause of death from cancer in women. With an annual incidence of approximately 297,790 new cases, it represents 31% of all new cancer diagnoses in women, and the mortality rate can reach up to 15% (1). In numerous developing countries and regions, the mortality rates are notably higher (2). BRCA is categorized into four subtypes based on molecular markers and immunohistochemistry scores: 1. Luminal A (LumA); 2. Luminal B (LumB); 3. HER-2 positive; 4. Triple-negative BRCA (TNBC), accounting for 12% to 24% of all BRCA cases (3, 4). In comparison to other BRCA subtypes, TNBC exhibits significant heterogeneity and aggressiveness, characterized by higher and earlier recurrence rates, elevated metastatic potential, and a poorer prognosis (5). Currently, effective therapeutic targets for TNBC are absent, with treatment primarily dependent on cytotoxic chemotherapy (6). Promising therapeutic advances have been made through the development of drugs (7, 8). However, the widespread use of these therapies is hindered by cost and additional constraints. Consequently, the identification of novel predictive and therapeutic biomarkers for BRCA, particularly TNBC, is essential to mitigate BRCA-related morbidity and mortality.

V-ATPases, highly conserved macromolecular enzymes, ubiquitously integrate into cellular and organelle membranes, regulating essential eukaryotic physiological processes. Primarily, they govern transmembrane proton pumping and intracellular compartment acidification to sustain pH homeostasis and facilitate intracellular membrane trafficking and protein degradation (9–11). These functions are critical for regulating cell signaling pathways, immune responses, and neurotransmitter release (12, 13). The eukaryotic V-ATPase is a large complex composed of 14 distinct subunits that are organized into two sectors: the peripheral domain (V1) and the central domain (V0) (14). Dysregulation of different subunits of V-ATPase results in diverse impacts on carcinogenesis and tumor progression. For example, V0a2 promotes tumor invasion in ovarian cancer cell lines, V0a3 influences melanoma metastasis, and both V0c and V1B2 exhibit elevated expression in Ewing’s sarcoma (15–17). Additionally, V-ATPase is intimately associated with BRCA cell tumorigenicity, invasion, and drug resistance (18–21).

ATPase H+ transporting V0 subunit d2 (ATP6V0D2), also called VMA6, encodes a V-ATPase subunit isoform. In a pan-tumor tissue microarray, ATP6V0D2 was overexpressed in various kinds of carcinomas, including kidney cancer, pancreas cancers and melanoma. Moreover, in KRAS mutation-driven cancers, ATP6V0D2 shows significantly overexpression at both mRNA and protein levels and intimately correlated with energy metabolism level, migration, and invasion (22). ATP6V0D2 has also been found to influence the development and progression of esophageal cancer and lung adenocarcinoma and is associated with the prognosis of patients (23, 24). Furthermore, ATP6V0D2 plays an important role in immune cells, especially in the functional regulation of macrophages (25), the tumor microenvironment, and the innate immune response. Affecting by key signaling pathways and molecules, ATP6V0D2 can regulate the polarization of macrophage as well as inflammatory cell infiltration, thereby affecting tumor progression (24) and immune responses (26). Nevertheless, its function and tumor immunity in BRCA remains uncertain.

Herein, we explored the expression of ATP6V0D2 across various cancers using public databases and evaluated its prognostic and diagnostic potential in BRCA Subsequently, we employed bioinformatics analysis to predict associated pathways, immune characteristics, and mutations related to ATP6V0D2. Finally, we conducted both *in vitro* and *in vivo* studies to examine the biological functions of ATP6V0D2 in TNBC. This study offers insights into BRCA molecular diagnosis and precision treatment.

## 2 Methods and materials

### 2.1 Database acquisition

The expression profiles of BRCA were retrieved from the University of California Santa Cruz (UCSC) database (https://xenabrowser.net/) along with the Cancer Genome Atlas (TCGA)-BRCA clinical information. For further analysis, a total of 1087 breast cancer (BC) samples and 113 normal breast tissues were obtained. Additionally, the BRCA dataset (GSE57297) was retrieved from the database of Gene Expression Omnibus (GEO) (https://www.ncbi.nlm.nih.gov/geo/).

### 2.2 Collection of pathological specimens

Unpaired BRCA (n=30) and normal breast (n=30) tissues for RNA isolation were collected from patients undergoing surgery at The Fourth Hospital of Hebei Medical University (Shijiazhuang, China) from 2023 October to December. No participants in the study had undergone any treatment before the surgical procedure. Paired breast cancer tissues (n=20) and para-tumor tissues (n=20) used for immunohistochemical (IHC), derived from paraffin-embedded specimens of BRCA patients, were obtained through the Department of Pathology at the Fourth Hospital of Hebei Medical University. The study participants were exclusively female, all diagnosed with invasive breast cancer. The age range of the participants was evenly distributed between 25 and 70 years. The research received approval from the Ethics Committee of the Fourth Hospital of Hebei Medical University.

### 2.3 ROC curves and survival analysis

ROC (Receiver operating characteristic) curve analysis to assess the diagnostic efficiency of ATP6V0D2 (27). The diagnostic accuracy was evaluated by determining the area under the curve (AUC), with a higher AUC value indicating better predictive ability. The correlation between ATP6V0D2 expression levels and prognosis was examined using the Kaplan-Meier log-rank test and the Cox proportional hazards model. Next, survival analysis and the corresponding curves were produced via the R package “survminer”. For each analysis, the most statistically significant cut-off for high vs. low ATP6V0D2 expression was determined by the surv_cutpoint function.

### 2.4 Identifying the differentially expressed genes (DEGs) and conducting enrichment analysis

Patients with BRCA from the TCGA were categorized into two cohorts based on their median ATP6V0D2 expression levels. The identification of differentially expressed genes (DEGs) was performed using the “DESeq2” R package. Then, functional enrichment analyses using Gene Ontology (GO) and Kyoto Encyclopedia of Genes and Genomes (KEGG) pathways were conducted employing the “clusterProfiler” R package. A threshold of adjusted P-value below 0.05 was set to indicate statistically significant enrichment. Additionally, Gene Set Enrichment Analysis (GSEA) was used to clarify the influence of ATP6V0D2 expression on possible cancer-related pathways.

### 2.5 Immune checkpoint, tumor immune infiltration, and gene mutation analysis

We utilized the Tumor Immune System Interactions and Drug Bank (TISIDB) database (http://cis.hku.hk/TISIDB/download.php) to assess the correlations between ATP6V0D2 expression in various cancers and immune checkpoints. Spearman’s rank correlation was calculated to examine the association between ATP6V0D2 and tumor-infiltrating immune cells in BRCA. The “ssGSEA” R package was employed to determine the levels of infiltration by ten distinct immune cell types and to compute their respective enrichment scores in BRCA (28). Additionally, the “maftools” R package was utilized to investigate the correlation between gene mutation profiles and the expression levels of ATP6V0D2.

### 2.6 RNA isolation and reverse transcription quantitative polymerase chain reaction (RT-qPCR)

Total RNA was extracted from both BRCA cells and tissue samples utilizing the TRIzol (Takara, Japan) then reverse transcribed with the TRUEscript RT MasterMix Kit (Aidlab, China) in accordance with the instructions. Subsequently cDNA samples were prepared and processed using the Go Taq^®^ SYBR Green qPCR Master Mix (10 μL) (Promega, China) for RT-qPCR. The human GAPDH gene served as a reference gene for normalization. The relative expression levels of RNAs were calculated using the 2^-ΔΔCt^ method. The related sequence of primers are presented in **Supplementary Table S2.**

### 2.7 Cell lines

MDA-MB-468 and MDA-MB-231, human breast cancer cell lines, were procured from Procell Life Science & Technology Co., Ltd. (Wuhan, China). They were grown in Dulbecco’s Modified Eagle Medium (DMEM) enriched with 10% Fetal Bovine Serum (FBS; Shanghai XP Biomed Co., Ltd., Shanghai, China) and 100 µg/mL penicillin/streptomycin (Beijing Solarbio Life Sciences Co., Ltd.). All cells were cultured in a sterile humidified incubator at 37°C with 5% CO_2_.

### 2.8 Histology and immunohistochemistry (IHC)

Mouse tumor specimens were preserved in a 10% formalin solution for 24 hours before being embedded in paraffin. Five-μm-thick sections were cut from the paraffin blocks and subjected to a series of procedures including deparaffinization, rehydration, antigen retrieval, and the quenching of endogenous peroxidase activity. To minimize non-specific interactions, the sections were pre-treated with a 10% solution of goat serum. They were then incubated with primary antibodies against ATP6V0D2 (ORIGENE, at a dilution of 1:500) and KI67 (ORIGENE, at a dilution of 1:300) at 4°C overnight. Afterward, the sections were exposed to secondary antibodies for 60 minutes. Color development was achieved using a DAB substrate, and the sections were finally stained with hematoxylin to visualize cell nuclei. Image J software was used to analyze protein staining.

### 2.9 Cell transfection

Transient siRNA knockdown was performed using GP-transfect-Mate following the instructions provided by the manufacturer. Transfection reagent, siRNAs targeting ATP6V0D2, and negative control siRNA were procured from Shanghai GenePharma Co. (Shanghai, China). For stable knockdown of ATP6V0D2 in MDA-MB-231, we carried out RNA interference using lentiviral vectors that contained validated shRNA sequences, with the assistance of polybrene. At the same time, random oligonucleotides were inserted into the same vector as a comparative control. After transfection the cell was selected with puromycin (2μg/ml). The shRNA sequence targeting human ATP6V0D2 and lentiviral packaging plasmids were also both obtained from Shanghai GenePharma Co. The sequences of the siRNA and shRNA are provided in **Supplementary Table S2.**

### 2.10 Western blot and antibodies

Proteins were isolated using a radio-immunoprecipitation assay buffer from Solarbio Co. (Beijing China), supplemented with 1% phenylmethylsulfonyl fluoride (PMSF) at low temperatures. The extracted proteins were subjected to sodium dodecyl sulfate-polyacrylamide gel electrophoresis (SDS-PAGE) and subsequently transferred to a 0.45 μm polyvinylidene fluoride (PVDF) membrane. Next, the membranes were blocked by rapid blocking solution (Epizyme Biotech, China) for 15 minutes before being incubated with primary antibodies specific for ATP6V0D2 (Abcam, diluted 1:500) and GAPDH (Proteintech, diluted 1:5000) overnight at 4°C. After cleaning the membranes with three washes, they were incubated with horseradish peroxidase- coupled secondary antibodies (SA00001-1 and SA00001-2, Proteintech, diluted 1:10000) at 37°C for 60 minutes. The detection of the immunoblots was achieved using a luminescent imaging device (Bio-Rad, USA) with an enhanced chemiluminescence detection kit. Quantification of protein expression intensities was performed using the ImageJ software.

### 2.11 Cell counting kit-8 (CCK-8) and colony proliferation assays

MDA-MB-468 and MDA-MB-231 cells (3 × 10^3^) were seeded into 96-well plates then cultured for 1 day. CCK-8 reagent (10 μl; MCE, MedChemExpress, Shanghai, China) was added to the wells every 24 hours for 5 days. After a 1-hour incubation at 37°C, the absorbance at 450 nm was recorded by a microplate reader (Tecan, Männedorf, Switzerland).

For colony proliferation assays, cells (2 × 10^3^) from various treatment groups were inoculated in a 6-well plate per well and incubated for 10 days in a complete medium. Visible punctate cell aggregates were established, fixed using paraformaldehyde, and stained with a 1% crystal violet solution. Colony assessment and counting were performed under an inverted microscope.

### 2.12 Transwell assay

The ability of cells to migrate and invade was assessed using 24-well Transwell systems with an 8.0 μm pore size (Corning, USA). A total of 4 × 104 cells from various treatment groups were seeded in the upper chamber with serum-free medium, while the bottom was filled with medium supplemented with 10% Fetal Bovine Serum (FBS). For invasion tests, the chamber’s upper surface was coated with Matrigel (BD Biosciences, Franklin Lakes, NJ, USA). The subsequent steps mirrored those for migration assays. After a 1 to 2 days period, chambers containing the migrated or invaded cells were fixed, stained, and then quantified and imaged in three randomly selected fields.

### 2.13 Scratch-healing assay

For the scratch healing assay, 3×10^5^ MDA-MB-468 or MDA-MB-231 cells were seeded into 6-well plates until reaching 80% convergence. A linear scratch was created in the uniform layer of cells with a sterile 10 μl pipette tip, followed by three washes with phosphate buffer solution. Subsequently, the cells were cultured in a serum-free environment, and collecting the images at 0 and 24-hour marks. The rates of wounded closure were determined by calculating the distance cells migrated, using the formula: Scratch healing rate (%) = [(scratch width at 0 hours - scratch width at 24 hours)/(scratch width at 0 hours)] × 100%.

### 2.14 Mouse xenograft tumor

Approval for conducting *in vivo* experiments was granted by the Institutional Animal Care and Use Committee (IACUC) at the Fourth Affiliated Hospital of Hebei Medical University. Ten female Balb/c-nude mice, each 4 weeks old and weighing between 15 and 18g, were sourced from Huafukang Bio-technology (Beijing, China). Following a 7-day acclimatization phase, they were randomly assigned to two groups, each consisting of 5 individuals, and 5 × 10^6^ MDA-MB-231 cells with or without ATP6V0D2 knockdown were subcutaneously inoculated into the right mammary fat pads of 5-week-old Balb/c-nude mice. Tumor size and volume were assessed every three days by a caliper. At the conclusion of the research, mice were humanely euthanized. Subsequently, the tumors were carefully removed, weighed, and prepared for additional analysis.

### 2.15 Statistical analysis

Statistical analyses were conducted using GraphPad Prism 8.0 and by R version 4.2.2. The results of experiments were shown as mean ± SD. For comparing two independent groups, unpaired Student’s t-tests and Wilcoxon rank sum tests were employed, whereas the Wilcoxon signed-rank test was utilized to assess variations in ATP6V0D2 expression within paired groups. The influences on patients’ DSS were assessed through Cox regression analyses. Log-rank test was used to assess the prognosis of cancer. Kaplan-Meier methodology was utilized to generate survival curves. Statistical correlations between two genes were quantified using Spearman Correlation analysis. *P*-values < 0.05 was considered as statistically significant.

## 3 Results

### 3.1 ATP6V0D2 was aberrantly expressed in pan-cancer

**Figure 1:**
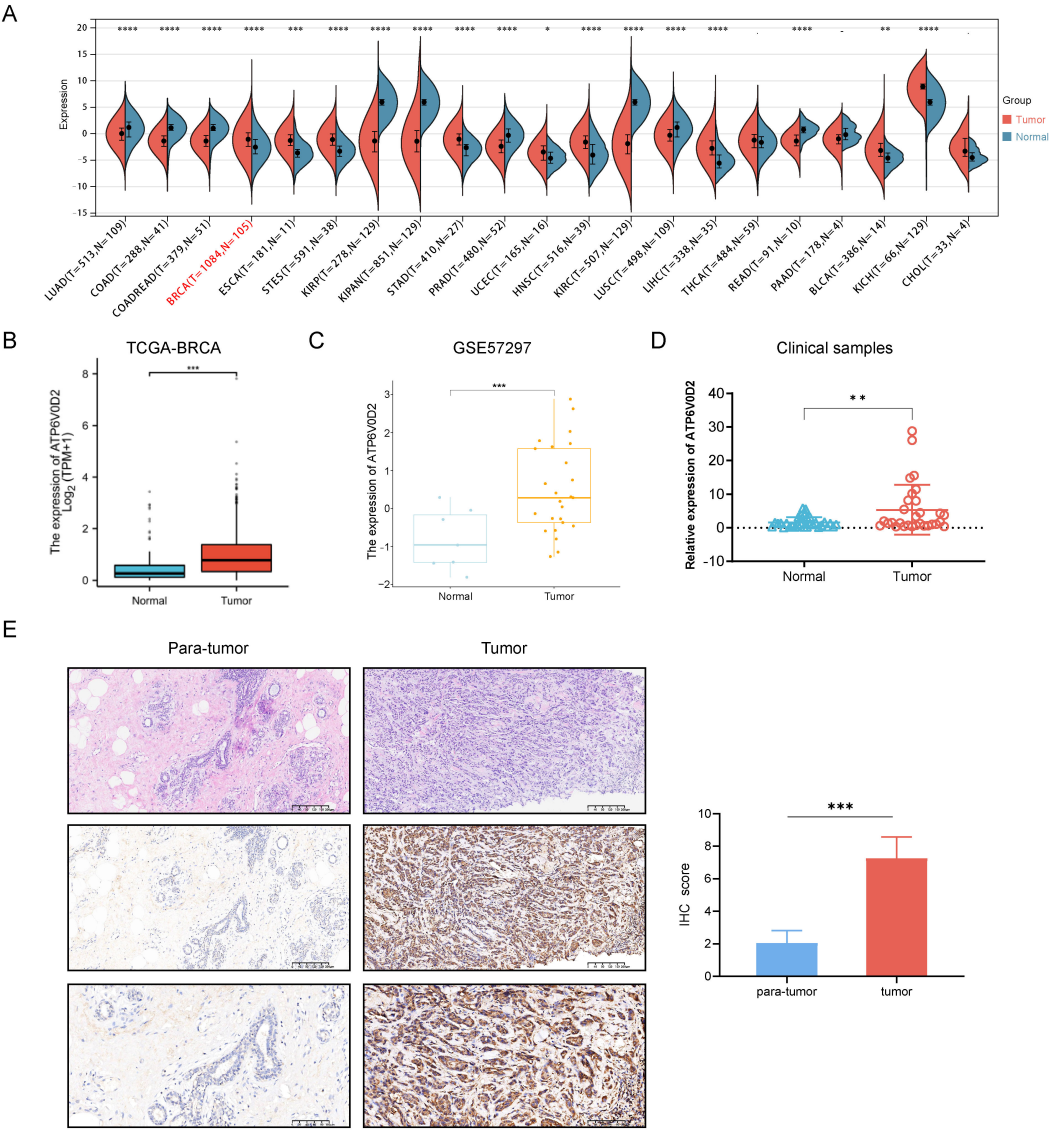
The expression level of ATP6V0D2 in patients with BRCA. **(A)** ATP6V0D2 expression in pan-cancer using TCGA RNA-seq data (Wilcoxon rank sum test). **(B)** ATP6V0D2 expression at the mRNA level in BRCA and normal tissues using TCGA-BRCA data (Wilcoxon rank sum test). **(C)** ATP6V0D2 expression at the mRNA level in BRCA and normal tissues using a GEO cohort (Wilcoxon rank sum test). **(D)** ATP6V0D2 expression in tumors (n=30) and normal tissues (n=30) in patients with BRCA assessed using RT-qPCR analysis (Unpaired student’s t−test). **(E)** Representative H&E and IHC images (100x and 200x magnification) demonstrated high expression of ATP6V0D2 in BRCA tissues (Wilcoxon signed-rank test); the score of ATP6V0D2 expression in IHC images is shown in the right part. (*p-value < 0.05; **p-value <0.01; ***p-value <0.001; ****p-value <0.0001).

First, we compared ATP6V0D2 expression between normal and tumor samples using data of TCGA. Results indicated significantly overexpression of ATP6V0D2 in BRCA, bladder urothelial carcinoma (BLCA), esophageal carcinoma (ESCA), cholangiocarcinoma (CHOL), stomach adenocarcinoma (STAD), and several other types of cancer compared to normal tissues. In contrast, prostate adenocarcinoma (PRAD), kidney renal clear cell carcinoma (KIRC), lung adenocarcinoma (LUAD), and colon adenocarcinoma (COAD) exhibited significantly reduced ATP6V0D2 expression compared to normal tissues. This analysis revealed that ATP6V0D2 expression levels were markedly aberrant in the most types of cancers (**Figure 1A**).

Subsequent analysis of ATP6V0D2 mRNA expression across TCGA revealed significant upregulation in BRCA tissues (**Figure 1B**). Consistent findings were observed in an independent GEO database (**Figure 1C**). RT-qPCR analysis further confirmed elevated ATP6V0D2 expression in unpair clinical BRCA tissues compared to thirty normal breast tissues (**Figure 1D**). Next, we performed IHC staining on paired primary BRCA tumor and para-tumor tissues. The analysis of IHC staining score also revealed a marked increase in the expression levels of the ATP6V0D2 protein within the breast cancer tissue samples (**Figure 1E**).

### 3.2 ATP6V0D2 expression was negatively correlated with prognosis in BRCA

**Figure 2:**
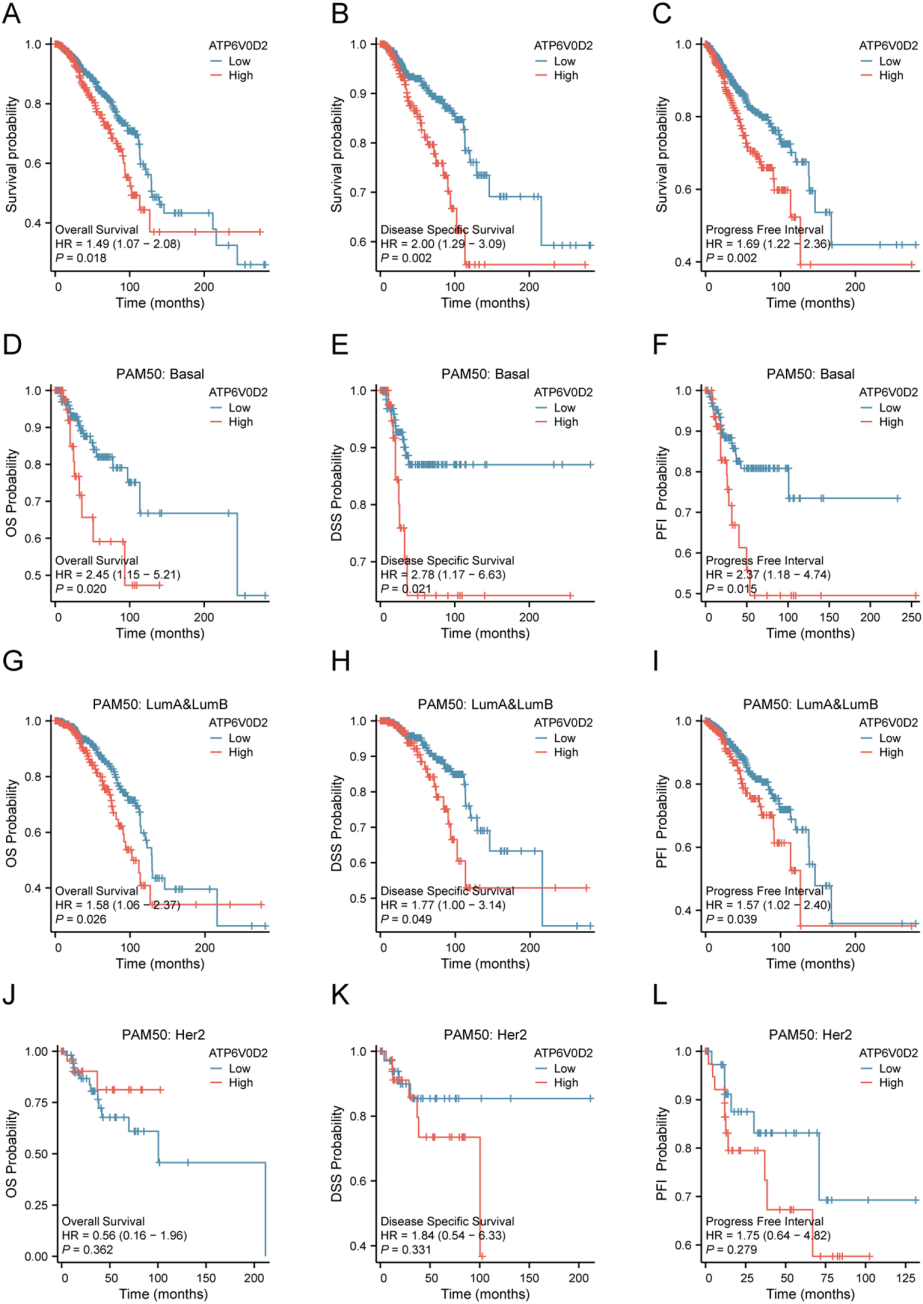
Relationship between ATP6V0D2 expression and prognosis of BRCA using the TCGA data. **(A)** Kaplan-Meier survival curve of overall survival (OS) in BRCA. **(B)** Kaplan-Meier survival curve of disease-specific survival (DSS) in BRCA. **(C)** Kaplan-Meier survival curve of progress-free interval (PFI) in BRCA. **(D-F)** Kaplan-Meier survival curve of OS, DSS, PFI in the basal BRCA subtype. **(G-I)** Kaplan-Meier survival curve of OS, DSS, PFI in LumA&LumB BRCA subtype. **(J-L)** Kaplan-Meier survival curve of OS, DSS, PFI in HER2 BRCA subtype (Log-rank test).

**Figure 3:**
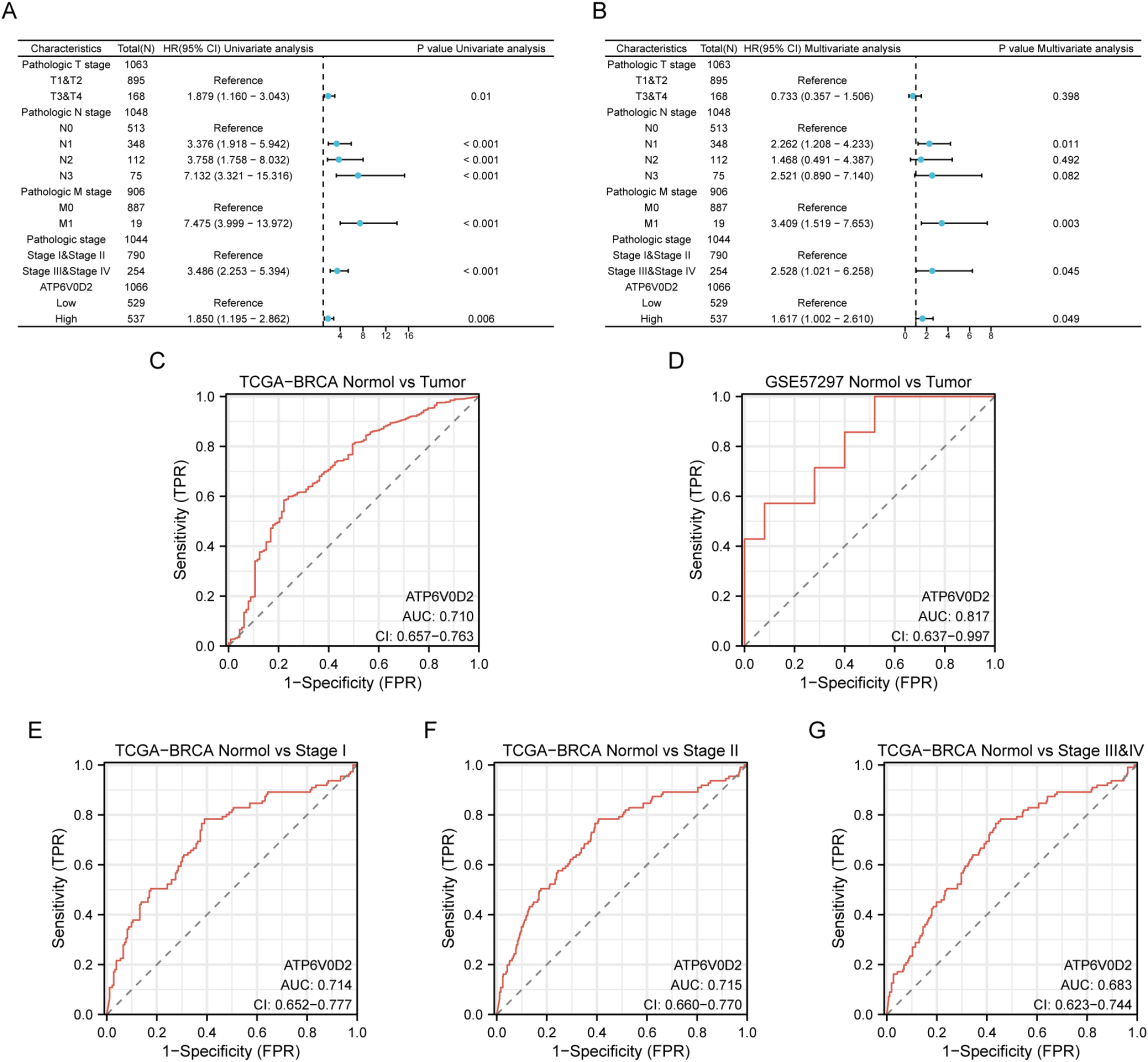
Potential clinical significance of ATP6V0D2 in patients with BRCA. **(A, B)** Univariate and multivariate Cox analyses of ATP6V0D2 in disease-specific survival and clinicopathological characteristics. **(C, D)** ROC curves of ATP6V0D2 expression in the TCGA-BRCA and GEO cohorts. **(E-G)** ROC curves of ATP6V0D2 expression in different pathological stages of the TCGA-BRCA cohort.

Given the distinct expression pattern of ATP6V0D2 in BRCA tissues compared to healthy tissues, we hypothesized its potential prognostic significance in BRCA. We evaluated the association between ATP6V0D2 levels and survival rates in BRCA patients, utilizing data from the TCGA and applying Kaplan-Meier estimates along with univariate Cox proportional hazards modeling for the assessment. Elevated ATP6V0D2 associated with significantly poorer overall survival (OS), disease-specific survival (DFS), and progression-free interval (PFI) (**Figures 2A–C**). Further subgroup analysis revealed that elevated ATP6V0D2 levels correlated with poorer outcomes in the LumA, LumB, and TNBC breast cancer subtypes, but not in HER2-enriched cases (**Figures 2D–L**). Additionally, Cox regression analysis indicated a substantial correlation between ATP6V0D2 levels and the likelihood of disease-specific survival in BRCA patients, persisting after accounting for various confounding factors in a multivariate setting (**Figures 3A, B**).

### 3.3 Diagnostic significance of ATP6V0D2 in BRCA

We utilized ROC curves to assess the diagnostic effectiveness of ATP6V0D2 mRNA expression levels in identifying BRCA tissues. The AUC metrics for ATP6V0D2 were respectively 0.710 and 0.817 in TCGA and GSE57297 cohorts (**Figures 3C, D**). Moreover, examining ATP6V0D2 levels across various stages of breast cancer in the TCGA dataset produced AUC values of 0.809 for stage I, 0.812 for stage II, and 0.837 for stage III (**Figures 3E–G**). This data implies that ATP6V0D2 could be a potential candidate for a diagnostic marker in BRCA patients.

### 3.4 Association of ATP6V0D2 with multiple cancer pathways revealed by enrichment analysis

**Figure 4:**
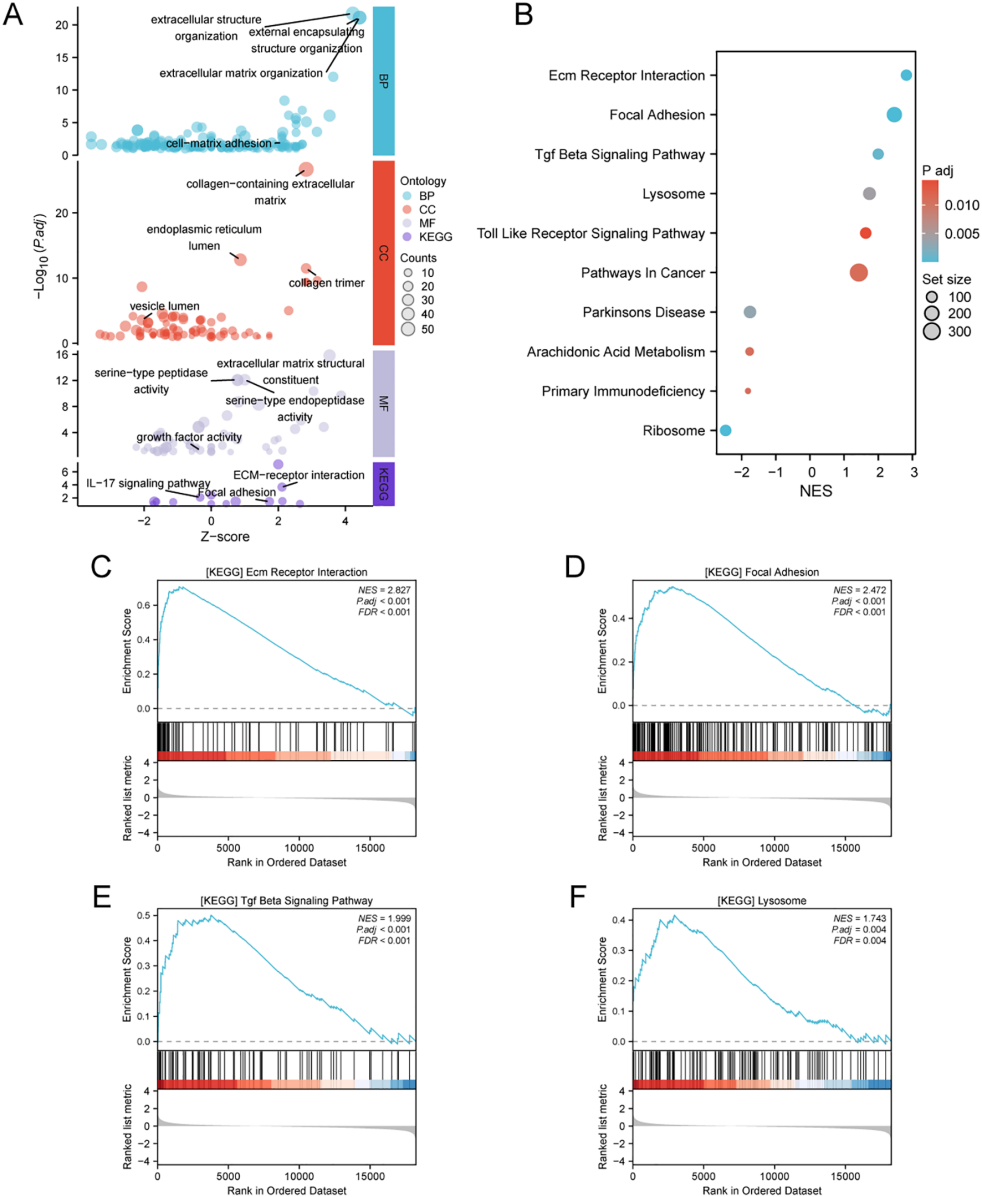
Analysis of differentially expressed genes (DEGs) related to ATP6V0D2 and functional enrichment of ATP6V0D2 in BRCA using analyses such as GO, KEGG, and GSEA. **(A)** GO (Gene Ontology) terms, including BP (Biological Process), MF (Molecular Function), CC (Cellular Component), and KEGG (Kyoto encyclopedia of genes and genomes) enrichment analysis of genes most strongly related to ATP6V0D2. **(B)** Dot plot of the DEGs identified through GSEA. The top four pathways enriched in GSEA analysis of ATP6V0D2 on KEGG were as follows: **(C)** ECM-receptor interaction, **(D)** Focal adhesion, **(E)** TGF beta signaling pathway, **(F)** Lysosome.

Patients with BRCA in the TCGA dataset were categorized into two cohorts based on the median ATP6V0D2 expression level. Subsequently, using the “DESeq2” R package, we identified DEGs, including 156 upregulated DEGs and 224 downregulated DEGs, which were further analyzed through GO and KEGG enrichment analyses to clarify ATP6V0D2’s potential functions in BRCA. According to the GO analysis, we found the DEGs were primarily enriched in GO terms, such as ‘extracellular matrix (ECM) organization’, ‘vesicle lumen’, and ‘growth factor activity’. KEGG analysis indicated significant associations of the DEGs with pathways encompassing ECM-receptor interaction and focal adhesion (**Figure 4A**). Additionally, GSEA was conducted to identify specific pathways associated with different expression groups (**Figure 4B**). The top four enriched pathways, based on normalized enrichment scores, were ‘Ecm-receptor interaction’, ‘tumor growth factor (Tgf)-beta pathway’, ‘focal adhesion’, and ‘lysosome’ (**Figures 4C–F**).

### 3.5 Correlations between ATP6V0D2 expression and immune checkpoints, immune cell infiltration

**Figure 5:**
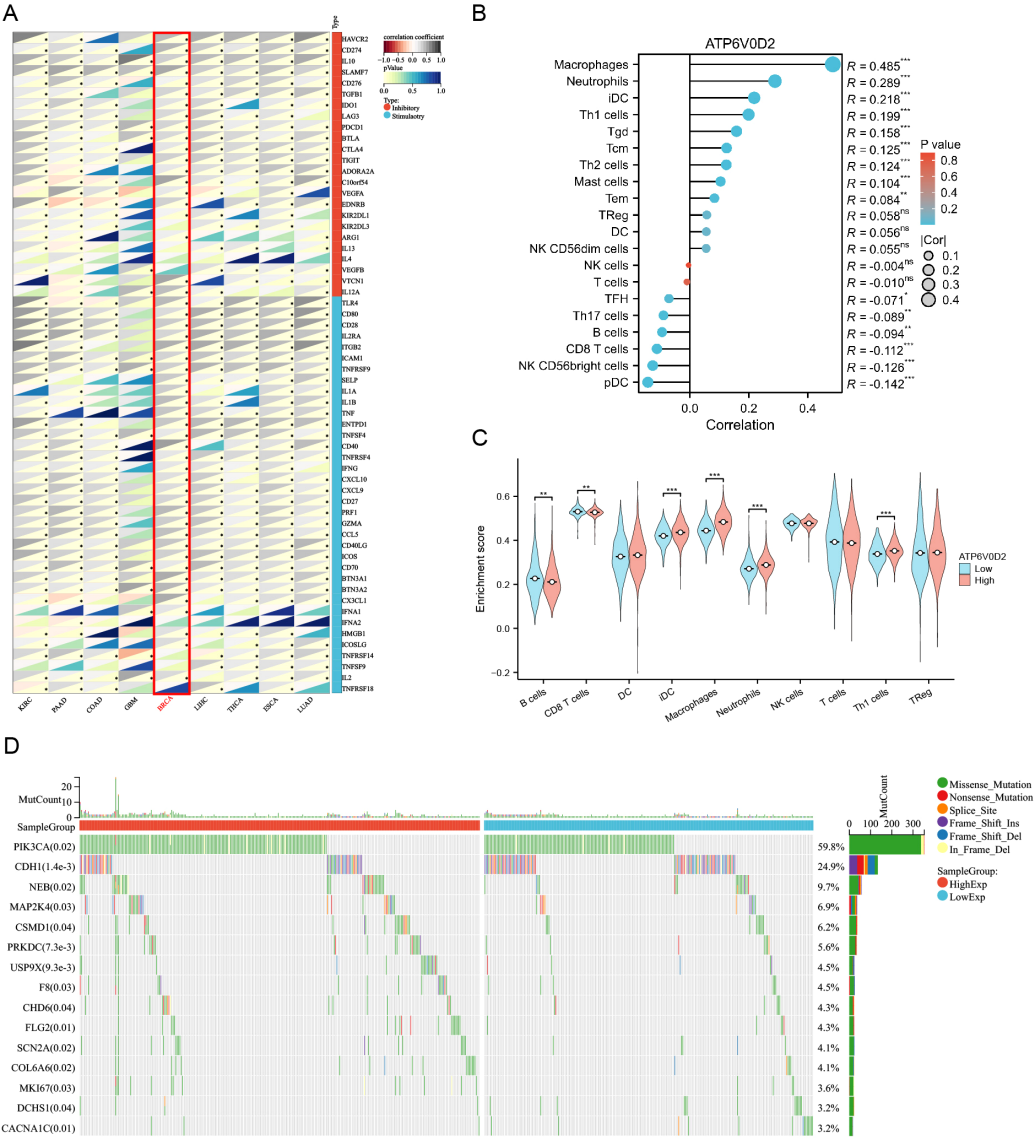
Correlation of immune checkpoints, immune infiltration analysis, and mutational analysis of ATP6V0D2 in BRCA. **(A)** Correlation between ATP6V0D2 expression and immune checkpoints in pan-cancer. The expression of genes related to immune checkpoints (Spearman correlation test). **(B)** Correlation between ATP6V0D2 expression and the relative abundance of immune cells in BRCA (Spearman correlation test). **(C)** Single-sample gene set enrichment analysis comparing the high and low ATP6V0D2 expression groups (Wilcoxon rank sum test). **(D)** Gene mutation analysis in high and low ATP6V0D2 groups. (*p-value < 0.05; **p-value <0.01; ***p-value <0.001) ns, no significance.

To investigate the immunological role of ATP6V0D2 across different cancers and assess its potential as an immunotherapy target, Spearman’s correlation analysis was performed in pan-cancer. In BRCA, ATP6V0D2 showed a positive association with multiple immune checkpoint proteins, such as CD274 known as PD-L1, CTLA4, and LAG3 (**Figure 5A**). The ssGSEA algorithm was utilized to assess the levels of lymphocyte infiltration within the tumor microenvironment (TME) (**Figure 5B**). Notably, ATP6V0D2 expression exhibited significant positive correlations with macrophage and neutrophil infiltration levels, while showing negative correlations with CD8+ T cells and plasmacytoid dendritic cells. Furthermore, the macrophage enrichment score was notably elevated in the group with high ATP6V0D2 expression, while the trend was reversed for CD8+ T and B cells (**Figure 5C**).

### 3.6 ATP6V0D2 is associated with gene mutation

To investigate how ATP6V0D2 expression levels relate to genetic mutations, a comparison of the mutational profiles was conducted for the high and low ATP6V0D2 expression cohorts. A summary of the top 15 mutated genes and the overall mutational landscape in BRCA was provided. In the high ATP6V0D2 expression group, genes including *PIK3CA, NEB, USP9X, COL6A6*, and *MKI67* were most susceptible to mutation (**Figure 5D**). Notably, PIK3CA mutations were associated with numerous cancers.

### 3.7 ATP6V0D2 knockdown suppresses the proliferation, invasion, and migration of TNBC cells

**Figure 6:**
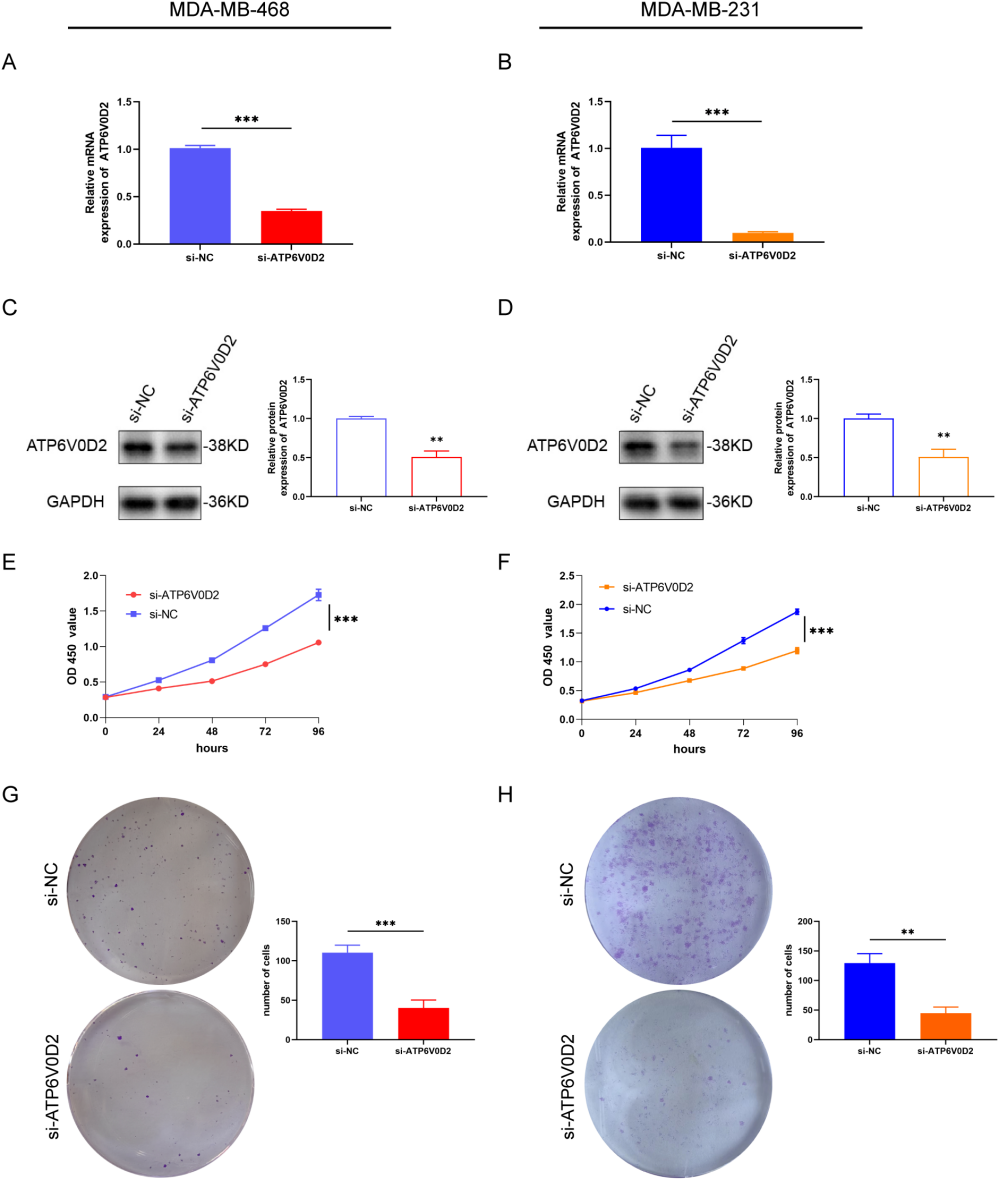
ATP6V0D2 knockdown suppressed TNBC cell proliferation *in vitro.* The transfection efficiency of ATP6V0D2 into MDA-MB-468 **(A, C)** and MDA-MB-231 cells **(B, D)** assessed using RT-qPCR and western blot. **(E, F)** CCK-8 assay of MDA-MB-468 and MDA-MB-231. **(G, H)** colony formation assay of MDA-MB-468 and MDA-MB-231 (Unpaired student’s t−test). (**p-value <0.01; ***p-value <0.001).

**Figure 7:**
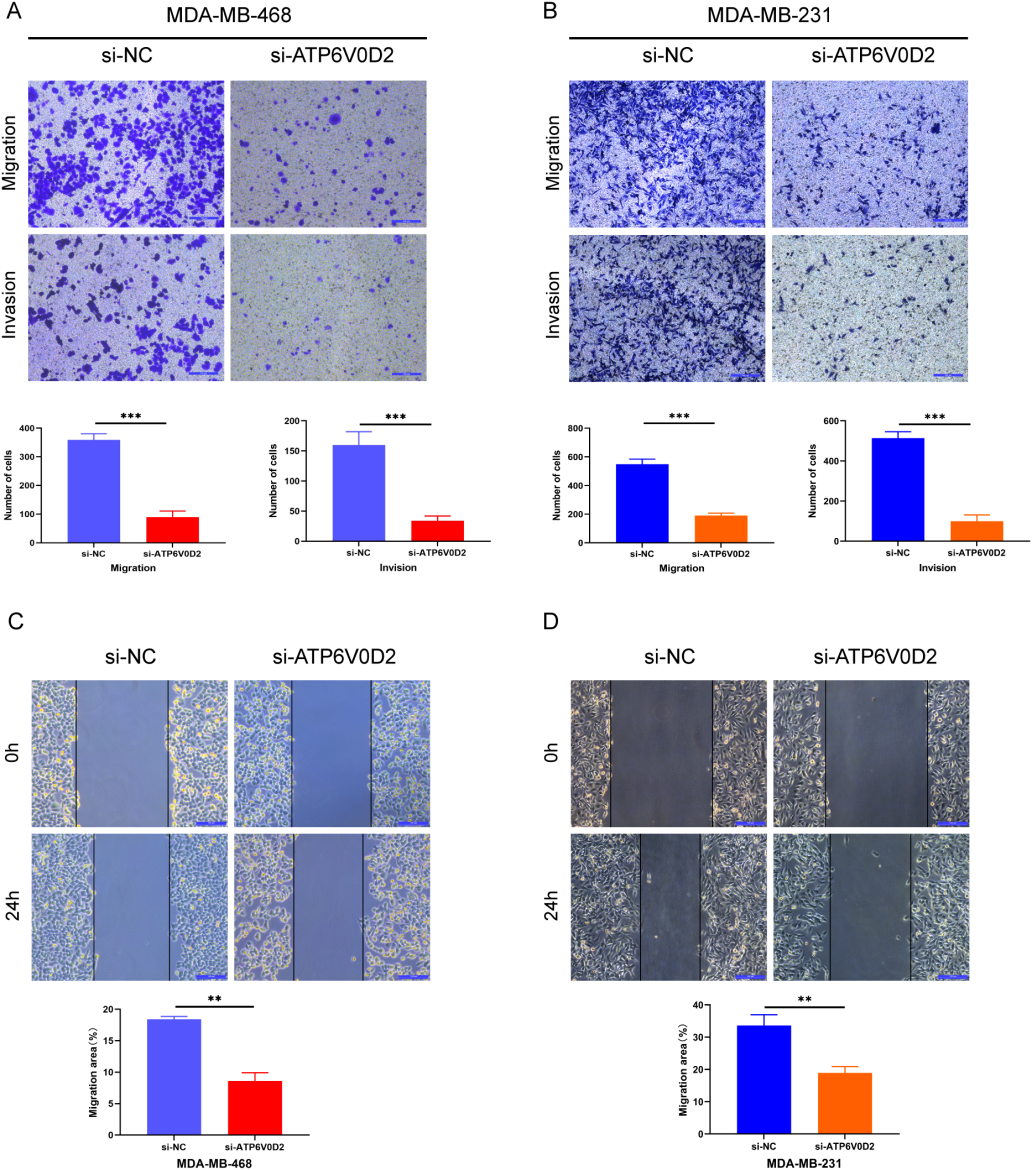
ATP6V0D2 knockdown inhibited TNBC cell migration and invasion *in vitro.* **(A, B)** MDA-MB-468 and MDA-MB-231 cell invasion and migration abilities assessed using Transwell assays. (Scale bar, 100 µm). **(C, D)** MDA-MB-468 and MDA-MB-231 cell migration ability after transfection assessed using wound healing assays (Unpaired student’s t−test). (Scale bar, 100 µm). (**p-value <0.01; ***p-value <0.001).

To evaluate the possible roles of ATP6V0D2 in TNBC, MDA-MB-468 and MDA-MB-231 cells were transfected with siRNA targeting ATP6V0D2. Successful transfection was confirmed by RT-qPCR and WB analysis, both of which confirmed significant downregulation of ATP6V0D2 at both mRNA and protein levels in MDA-MB-468 (**Figures 6A, C** and MDA-MB-231 (**Figures 6B, D**) cells. CCK-8 assays demonstrated that silencing ATP6V0D2 reduced cellular growth in both lines (**Figures 6E, F**), which was further confirmed by clone formation assays (**Figures 6G, H**). Moreover, Transwell and scratch closure assays were performed to evaluate the impact of ATP6V0D2 on the invasive and migratory capabilities of TNBC cells. ATP6V0D2 knockdown in MDA-MB-468 cells suppressed cell invasion and migration (**Figures 7A, C**), and analogous findings were noted in MDA-MB-231 cells (**Figures 7B, D**).

### 3.8 Suppressing ATP6V0D2 impedes the growth of mouse xenograft tumor

**Figure 8:**
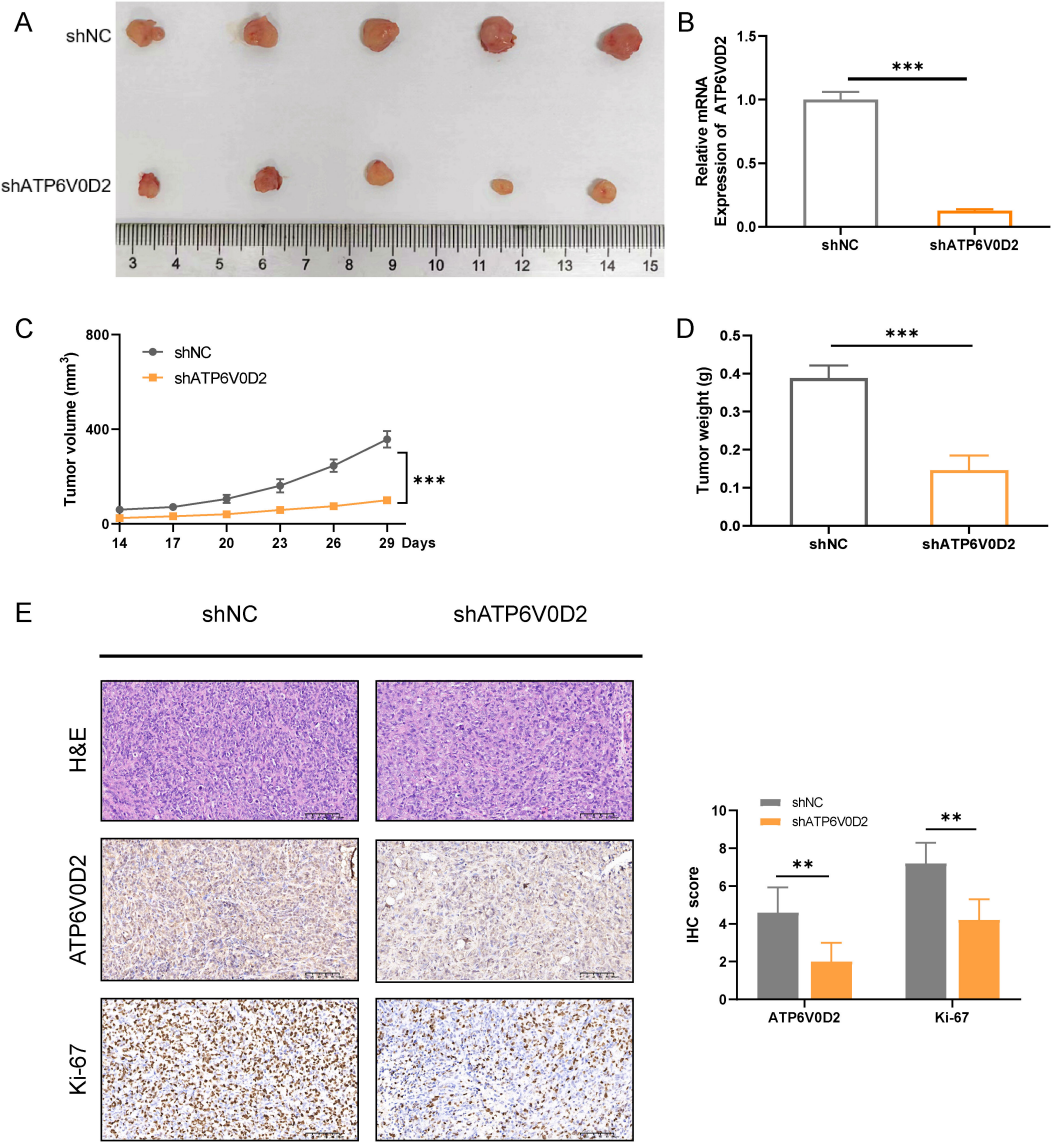
Suppressing ATP6V0D2 inhibit the growth of TNBC *in vivo.* **(A)** Images of xenograft tumors from nude mice. **(B)** The ATP6V0D2 mRNA level **(C)** volume, and **(D)** weight of xenograft tumors (Unpaired student’s t−test). **(E)** Representative H&E and IHC images of each group of xenograft tumors along with the IHC scoring right side (Unpaired student’s t−test). (**p-value <0.01; ***p-value <0.001).

To delve deeper into how ATP6V0D2 influences the progression of TNBC *in vivo*, we successfully established the MDA-MB-231 cell line with stable knockdown of ATP6V0D2, along with a negative control, using lentiviral transduction. We then injected different groups of cells into the mammary fat pad of female Balb/c nude mice. We extracted RNA from tumors in nude mice and determined that ATP6V0D2 was silenced and found that knockdown of ATP6V0D2 resulted in markedly smaller tumor volumes and weights compared to the control group (**Figures 8A–D**). Additionally, immunohistochemistry revealed that ATP6V0D2 knockdown resulted in reduced Ki67 expression levels (**Figure 8E**). Collectively, these findings provide compelling evidence that silencing ATP6V0D2 inhibits TNBC tumor growth *in vivo*.

## 4 Discussion

Despite advancements in diagnostic technology and development of novel drugs, BRCA continues to pose significant challenges in the field of oncology. Although numerous molecular factors have been associated with the progression of BRCA, reliable prognostic markers, particularly for TNBC, are still absent in clinical practice. This study investigated ATP6V0D2 expression across various cancers and assessed its prognostic significance in BRCA, along with its potential diagnostic implications. The relationship between ATP6V0D2 expression, immune characteristics, and gene mutations was also explored. Additionally, the potential functions of ATP6V0D2 in TNBC cells and mice were examined, aiming to identify a novel biomarker for BRCA diagnosis, prognosis, and therapeutic interventions.

In the present study, we found significant upregulation of ATP6V0D2 in nine out of 21 human cancer tissues, including BRCA from TCGA and GEO datasets. Similar result was confirmed in the mRNA level of ATP6V0D2 in BRCA clinical samples compared with normal breast tissues. Kaplan-Meier curve and univariate analyses revealed that elevated ATP6V0D2 expression associated with shorter OS, DSS, and PFI, particularly in TNBC. Furthermore, multivariate analysis established ATP6V0D2 as an independent prognostic factor for BRCA-related survival outcomes, suggesting its potential as a prognostic biomarker. ATP6V0D2 has also been linked to the development of a range of cancers. In KIRC, it was found to be more highly expressed in normal kidney tissues than in renal cancer tissues, suggesting its possible use as a biomarker for KIRC (29). Furthermore, ATP6V0D2 was markedly upregulated in esophageal cancer, where it was associated with enhanced proliferative and metastatic abilities of esophageal cancer cells (23). ATP6V0D2 also exhibited elevated expression in gastric cancer, whereas it was significantly downregulated in colon cancer (30). In lung adenocarcinoma, ATP6V0D2 was identified as essential for creating a lactate-enriched tumor microenvironment. It facilitates pro-tumoral activities in tumor-associated macrophages through hypoxia-independent regulation of hypoxia-inducible factor-2α (24). These results indicate significant functions for ATP6V0D2 in the development of multiple cancers. ATP6V0D2 also showed promising diagnostic efficacy across different cohorts, as evidenced by ROC curve analyses, with significant differences in AUC values between normal tissues and various tumor stages. This suggests ATP6V0D2 as a diagnostic biomarker for BRCA. However, precise biological role of ATP6V0D2 in BRCA remains unexplored. Therefore, a series of functional experiments, such as CCK-8 assays, colony formation assays, scratch healing assays, and transwell assays, were conducted in TNBC cells. The results indicated that ATP6V0D2 promotes proliferation, migration, and invasion abilities of TNBC cells, furthermore we verified ATP6V0D2 promotes tumor growth *in vivo* experiments, suggesting the function of ATP6V0D2 as an oncogene.

Further functional enrichment analysis revealed a correlation between elevated ATP6V0D2 level in BRCA and cancer progression. Enriched pathways in the high ATP6V0D2 expression group included ‘Ecm Receptor Interaction’, ‘Focal Adhesion’, ‘Tgf Beta Signaling Pathway’, ‘Lysosome’, and ‘Toll-Like Receptor Signaling Pathway’. The extracellular matrix (ECM), crucial for providing support and environment to tissues, plays a key role in initiating tumor formation, invasiveness, and the spread of cancer cells (31). The formation of adhesive interactions between tumor cells and their surrounding ECM is critical for migration (32). Additionally, proteins such as ATG9B facilitate colorectal cancer spreads by promoting the formation of focal adhesions (33). Interrupting the signaling pathways of Src and focal adhesion kinase (FAK), triggered by collagen IV, has been demonstrated to inhibit ECM-mediated invasion in TNBC that is related to chemotherapy (34). The TGF beta signaling pathway, regulating key cellular processes, is dysregulated in cancer (35). This pathway has been linked to osteolytic metastasis in BRCA (36), with studies emphasizing its role in tumorigenesis and cell proliferation (37). Previous research has established that ATP6V0D2 is intricately linked to alterations in the extracellular matrix and plays a role in fostering esophageal cancer invasion and metastasis by modulating the epithelial-mesenchymal transition (EMT) pathway (23). Based on our results of functional enrichment analysis, we propose the hypothesis that ATP6V0D2 may enhance the invasive and metastatic capabilities of breast cancer via the EMT pathway. ATP6V0D2 is also likely to enhance the proliferation and metastatic potential of breast cancer cells through the activation of the TGF and FAK signaling pathway or by preventing the deactivation of those pathways. Targeting ATP6V0D2 could potentially suppress the activity of these pathways, thereby inhibiting the progression of breast cancer.

The TME (tumor microenvironment), originating from adjacent mesenchymal stroma, comprises diverse tissues and numerous cell types, fostering a nurturing environment for tumorigenesis (38). This system includes an array of different elements such as mesenchymal cells, immune infiltrates, inflammatory cells, blood vessel lining cells, fat cells, and connective tissue cells, all crucial components influencing tumor formation, metastasis, and treatment resistance (39). Immune cell infiltration is a key factor influencing the effect of immunotherapy and patient outcomes. This study first established a relationship between ATP6V0D2 expression and the TME through bioinformatic analysis. Our findings revealed an inverse relationship between ATP6V0D2 levels and both CD8+ T cells and plasmacytoid dendritic cells, while a positive correlation was observed with macrophages. Notably, CD8+ T cells and plasmacytoid dendritic cells are pivotal within the tumor microenvironment and have been linked to favorable clinical outcomes (40). Tumor-associated macrophages (TAMs) also play a crucial role and have been demonstrated to promote tumor expansion by facilitating tumor vascularization through expressing of vascular endothelial growth factor in the TME (41). Additionally, research indicates that the presence of tumor-infiltrating lymphocytes significantly influences the clinical outcomes of immunotherapy for individuals suffering from melanoma and other types cancer (42). We hypothesized that ATP6V0D2 expression might modulate the proportion between M1 and M2 macrophages within the tumor microenvironment (TME). Furthermore, ATP6V0D2 could potentially alter the phenotype, density, and spatial distribution of immune cell infiltrates by modulating the extracellular matrix. This could have a profound impact on patient survival and responsiveness to immunotherapeutic interventions. The modulation of immune cell infiltration could significantly influence both survival rates and the efficacy of immunotherapy in breast cancer patients. However, further clinical research is imperative to verify these conjectures. Considering the elevated expression of ATP6V0D2 in breast cancer tissues and its significant association with the immune microenvironment, measuring ATP6V0D2 levels in BRCA tissues could be instrumental in forecasting patient outcomes and informing the development of targeted immunotherapy strategies.

Immune checkpoints serve as inhibitors of the immune system, important for self-tolerance and regulation of immune responses in peripheral tissues. However, tumors exploit these inhibitory pathways to evade immune cell-mediated killing. Blocking immune checkpoints holds promise as a strategy to trigger the immune system’s fight against cancer (43). In neoadjuvant therapy for TNBC, the combination of chemotherapy and immunotherapy has acquired more favorable outcomes (44). Nevertheless, there remains a subset of patients who exhibit limited responsiveness to immunotherapy, thereby failing to achieve the desired therapeutic outcomes. Enhancing the responsiveness of immunotherapy and accurately forecasting its effect on cancer patients is of paramount importance, and this has indeed become a pivotal area of focus in recent years. Prior research has revealed that GNPNAT1 expression levels, as determined by the TIDE algorithm, exhibit a positive correlation with immune checkpoint activity (45). However, GNPNAT1 levels show a negative correlation with the therapeutic outcomes of immunotherapy in TNBC. Evaluating the relationship between ATP6V0D2 and immune checkpoints may not only improve the efficacy of immune checkpoint inhibitors-related drugs but also serves as a promising predictive biomarker for immunotherapy. Patients exhibiting elevated immune checkpoint expression are more likely to benefit from immunotherapy. It was found that ATP6V0D2 levels were directly linked to higher expressions of immune checkpoints including CD274(PD-L1), CTLA4, TIGIT, and HAVCR2, across various tumors. We speculate ATP6V0D2 is posited to enhance the expression of immune checkpoints via specific signaling pathways. Consequently, the combined targeting of ATP6V0D2 with immunosuppressive agents could potentially enhance the potency of immunotherapy, thereby extending its benefits to a broader range of patients with advanced breast cancer, especially TNBC patients. Hence, ATP6V0D2 holds substantial promise as a therapeutic target for the development of adjunctive medications aimed at bolstering the effectiveness of immunotherapy. Moreover, we analyzed gene mutations in different ATP6V0D2 expression groups and found significant mutations in genes such as PI3KCA, CDH1, and MKI67. Notably, multiple PIK3CA mutations are correlated with reduced overall survival compared to wild-type PIK3CA in BRCA (46). Targeting ATP6V0D2 may offer novel therapeutic avenues for BRCA by modulating genes that participate in the PI3K-AKT signaling pathway.

Based on our understanding, this research appears to be the first exploration of ATP6V0D2 in BRCA. Nevertheless, it is essential to recognize some constraints. Firstly, the sample size of clinical specimens may not be sufficient, potentially leading to biased results. Secondly, the associations between ATP6V0D2 mRNA level and numerous immune cells, including their markers, might be affected by discrepancies in the sequencing data from public databases. Consequently, additional sequencing data is essential for confirmation. Lastly, the clinical application of these findings hinges on a deeper comprehension of the behind mechanisms that need to be explored. Therefore, additional high-quality researches with big sample are urgently needed to validate these conclusions.

## 5 Conclusion

In summary, this study explored the significance of ATP6V0D2 and its role in predicting outcomes in breast cancer. Elevated ATP6V0D2 levels are observed in breast cancer tissue samples and it promotes malignant progression of BRCA *in vitro* and *in vivo*. Additionally, a correlation has been observed between ATP6V0D2, immune characteristics, gene mutation. These findings collectively highlight the necessity for further research into ATP6V0D2’s function in breast cancer, particularly its potential as a prognostic marker and its treatment implications.

## Data Availability

The original contributions presented in the study are included in the article/**Supplementary Material**. Further inquiries can be directed to the corresponding author.
