# Supplementary material for: The role of ATP6V0D2 in breast cancer: associations with prognosis, immune characteristics, and TNBC progression
Source: Front Oncol. 2024 Nov 29;14:1511810. doi: 10.3389/fonc.2024.1511810 (PMC11638046; doi:10.3389/fonc.2024.1511810)
Supplement: Supplementary file 1 [file DataSheet1.docx]

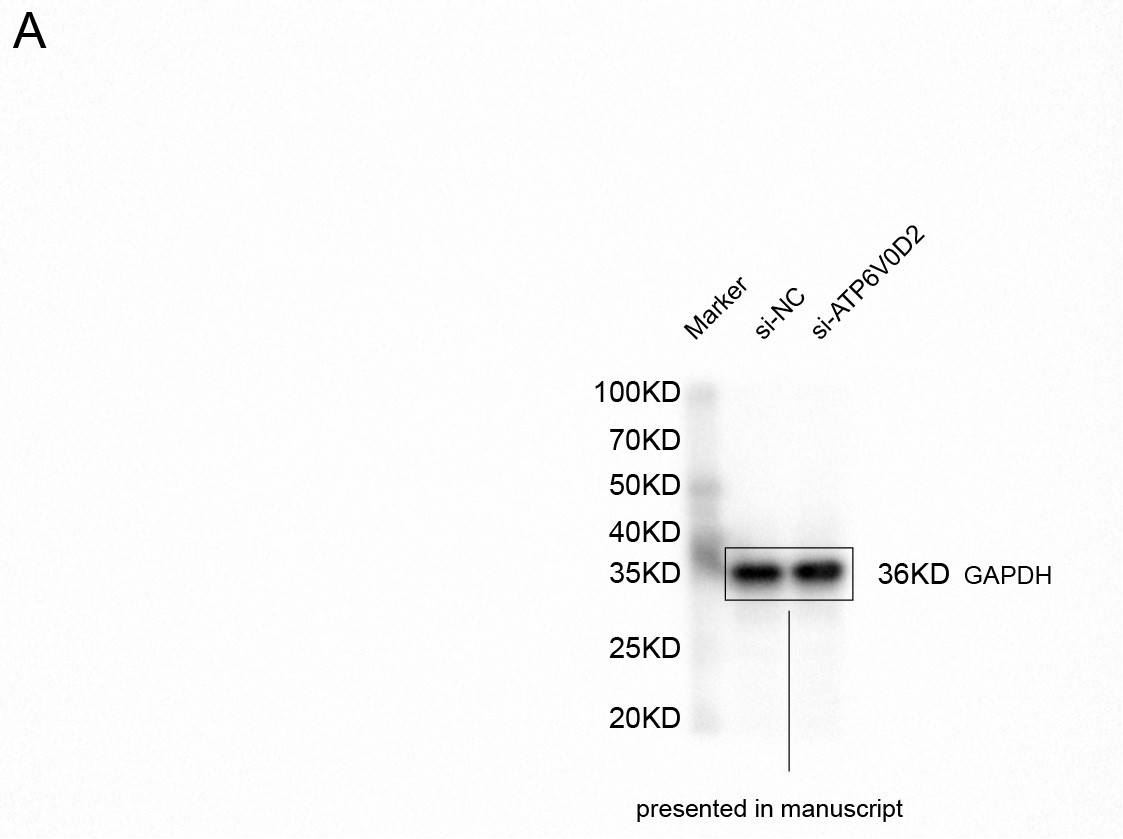


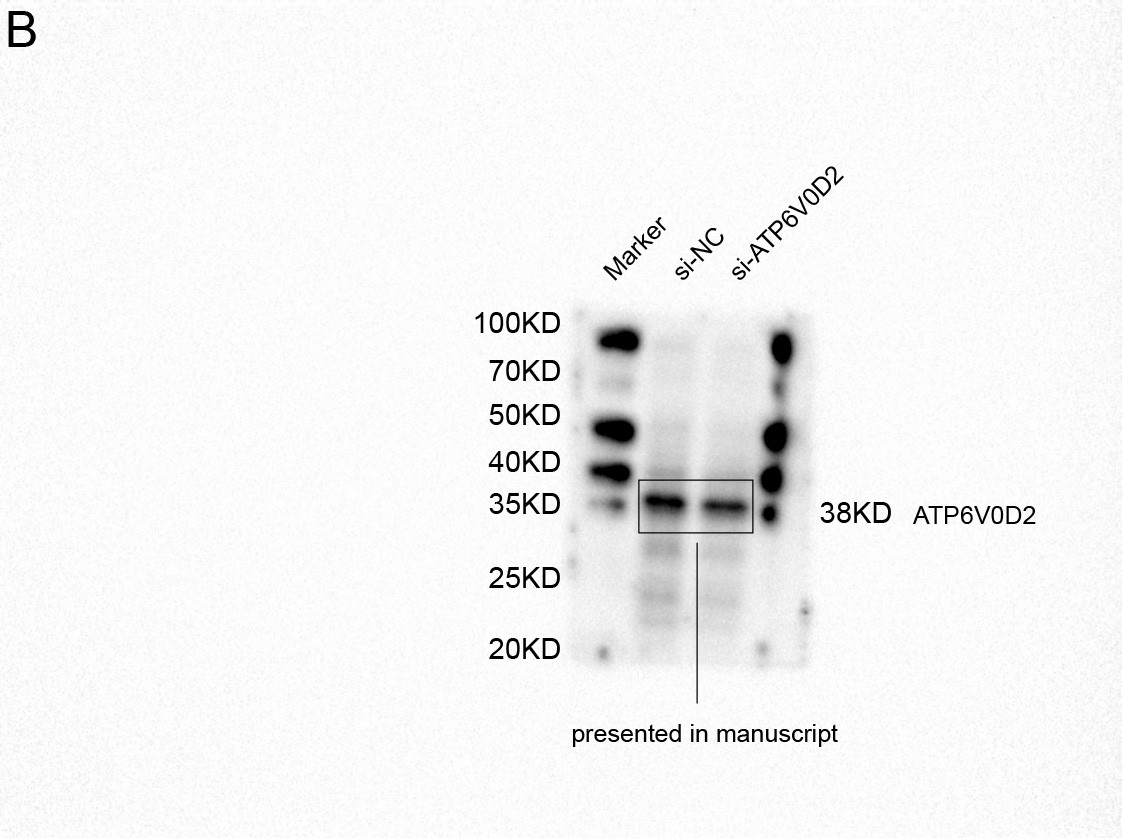


**Supplementary Figure S1:** The original gel of MDA-MB-468 cells transfected with siRNA represent the GAPDH (A) and ATP6V0D2 (B) expressions.


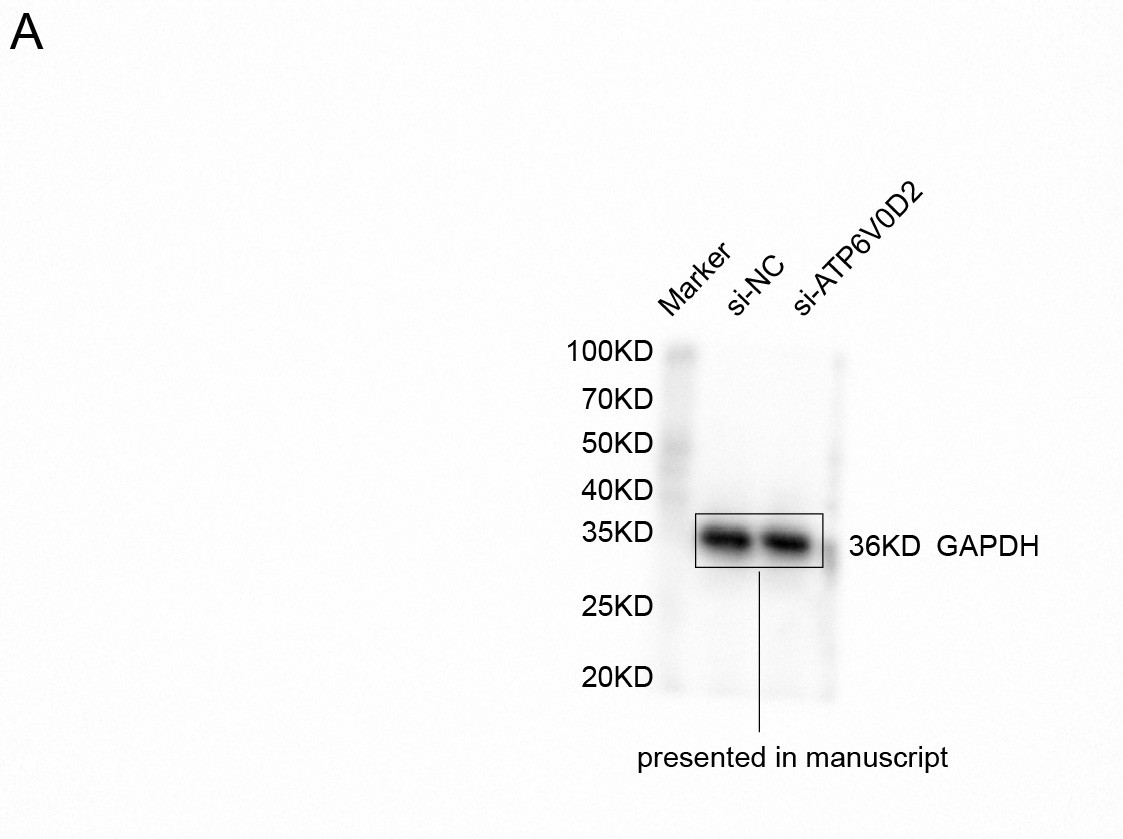


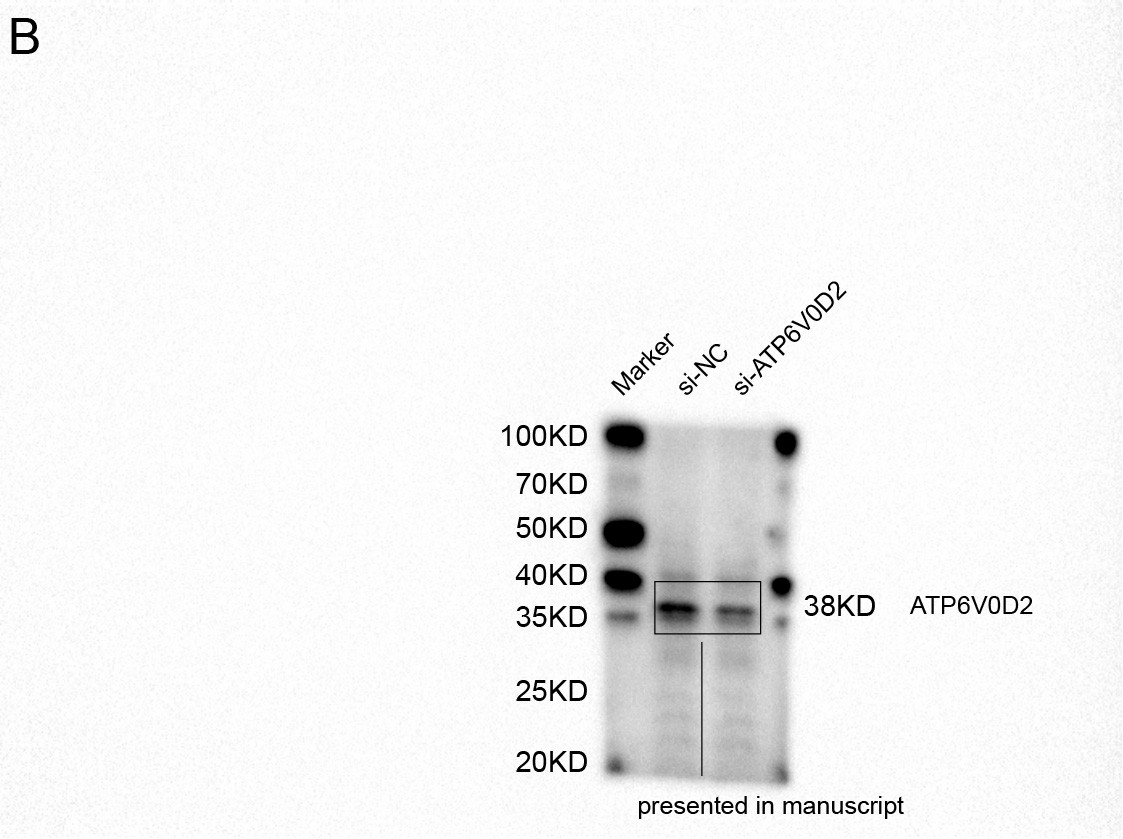


**Supplementary Figure S2:** The original gel of MDA-MB-231 cells transfected with siRNA represent the GAPDH (A) and ATP6V0D2 (B) expressions.

**Supplementary Table S1** Univariate and Multivariate analysis with ATP6V0D2 and breast cancer predictors

| Characteristics | Total(N) | Univariate analysis | |  | Multivariate analysis | |
| --- | --- | --- | --- | --- | --- | --- |
|  |  | Hazard ratio (95% CI) | P value |  | Hazard ratio (95% CI) | P value |
| Pathologic T stage | 1,063 |  |  |  |  |  |
| T1&T2 | 895 | Reference |  |  | Reference |  |
| T3&T4 | 168 | 1.879 (1.160 - 3.043) | **0.010** |  | 0.733 (0.357 - 1.506) | 0.398 |
| Pathologic N stage | 1,048 |  |  |  |  |  |
| N0 | 513 | Reference |  |  | Reference |  |
| N1 | 348 | 3.376 (1.918 - 5.942) | **< 0.001** |  | 2.262 (1.208 - 4.233) | **0.011** |
| N2 | 112 | 3.758 (1.758 - 8.032) | **< 0.001** |  | 1.468 (0.491 - 4.387) | 0.492 |
| N3 | 75 | 7.132 (3.321 - 15.316) | **< 0.001** |  | 2.521 (0.890 - 7.140) | 0.082 |
| Pathologic M stage | 906 |  |  |  |  |  |
| M0 | 887 | Reference |  |  | Reference |  |
| M1 | 19 | 7.475 (3.999 - 13.972) | **< 0.001** |  | 3.409 (1.519 - 7.653) | **0.003** |
| Pathologic stage | 1,044 |  |  |  |  |  |
| Stage I&Stage II | 790 | Reference |  |  | Reference |  |
| Stage III&Stage IV | 254 | 3.486 (2.253 - 5.394) | **< 0.001** |  | 2.528 (1.021 - 6.258) | **0.045** |
| ATP6V0D2 | 1,066 |  |  |  |  |  |
| Low | 529 | Reference |  |  | Reference |  |
| High | 537 | 1.850 (1.195 - 2.862) | **0.006** |  | 1.617 (1.002 - 2.610) | **0.049** |

**Supplementary Table S2** List of primers sequences

| Experiment types | Gene | Primers |
| --- | --- | --- |
| RT-qPCR | ATP6V0D2 | F:5’- GGTGATGTCACAGCAGAAGTTATG -3’ |
|  |  | R:5’- CCTCAGGATAGAGTTTGCCGAAG -3’ |
|  | GAPDH | F:5’- GTCTCCTCTGACTTCAACAGCG -3’ |
|  |  | R:5’- ACCACCCTGTTGCTGTAGCCAA -3’ |
| si-RNA |  |  |
|  | si-NC | S: 5′- UUUUCCGAACGUGUCACGUTT -3′ |
|  |  | AS: 5′- ACGUGACACGUUCGGAGAATT -3′ |
|  | si-ATP6V0D2 | S: 5′- GCGGAUCAUUACGGAGUAUTT -3′ |
|  |  | AS: 5′- AUACUCCGUAAUGAUCCGCTT -3′ |
|  |  |  |
| sh-RNA | sh-NC | 5′- TTCTCCGAACGTGTCACGT -3′ |
|  | sh-ATP6V0D2 | 5′- GCGGATCATTACGGAGTAT -3′ |

F: Forward primer; R: Reverse primer.

S: sense strand; AS: antisense strand.

**Histology and immunohistochemistry (IHC)**

Evaluation system for the IHC score: The immunoreactive score (IRS) gives a range of 0–9 as a product of multiplication between the positive cell proportion (score 0–4) and staining intensity (score 0–3). The score criteria for the proportion of positive cells were: ≤25% was 1 point, 26%-50% was 2 points, 51%-75% was 3 points, and >75% was 4 points. According to the intensity of staining, 1 point is scored for unstained, 2 points for light brown and yellow, 3 points for obvious brown and yellow, and 4 points for dark brown.
